# Electrostatic forces drive poleward chromosome motions at kinetochores

**DOI:** 10.1186/s13008-016-0026-1

**Published:** 2016-10-28

**Authors:** L. John Gagliardi, Daniel H. Shain

**Affiliations:** Departments of Physics and Biology, Rutgers The State University of New Jersey, Camden, NJ 08102 USA

**Keywords:** Mitosis, Ndc80/Hec1, Electrostatics, Chromosome, Motility

## Abstract

**Background:**

Recent experiments regarding Ndc80/Hec1 in force generation at kinetochores for chromosome motions have prompted speculation about possible models for interactions between positively charged molecules at kinetochores and negative charge at and near the plus ends of microtubules.

**Discussion:**

A clear picture of how kinetochores and centrosomes establish and maintain a dynamic coupling to microtubules for force generation during the complex motions of mitosis remains elusive. The current paradigm of molecular cell biology requires that specific molecules, or molecular geometries, for force generation be identified. However, it is possible to explain several different mitotic motions—including poleward force production at kinetochores—within a classical electrostatics approach in terms of experimentally known charge distributions, modeled as surface and volume bound charges interacting over nanometer distances.

**Conclusion:**

We propose here that implicating Ndc80/Hec1 as a bound volume positive charge distribution in electrostatic generation of poleward force at kinetochores is most consistent with a wide range of experimental observations on mitotic motions, including polar production of poleward force and chromosome congression.

## Background

Current thought on mitotic events is shifting somewhat to a more electrostatics-based framework, perhaps in line with theoretical predictions made over a decade ago [1, 2]. For example, electrostatic interactions at kinetochores between negatively charged microtubule plus ends and positive charge in kinetochores have been proposed for microtubule-chromosome binding [3] and chromosome motility during mitosis [4]. As is the case for most of the papers in this area, the other papers cited here focus on force generation at kinetochores within a molecular biology approach. The 2006 paper by Liu and Onuchic utilizes a very different (from the present paper) electrostatics-based approach [5]. Many currently favored models for chromosome motions involve interactions that are fundamentally electrostatic, including the so-called “mechanical” mechanisms for chromosome motility based on protofilament end splaying. Coupling molecules and molecular structures have been suggested to convert the progressive splaying (i.e., arching out into a “ram’s horn” configuration) of disassembling microtubule protofilaments into poleward force generation for chromosome movements [6]. In later versions of these models, the splaying tendency of GDP tubulin protofilaments to curve in this manner provides a “power stroke” that pulls on centromeric chromatin through kinetochore fibrils [7]. Other models utilize ring-like coupling kinetochore Dam1/DASH complexes for this coupling [8–11].

Regarding Ndc80/Hec1, the subject of the present work, a 2015, J. Cell Biol. paper by Zaytsev et al. [12] proposes that the N-terminal binding domain of CENPF interacts with curled oligomers of tubulin contributing to firm bonds between kinetochores and the flared end of dynamic microtubules. A 2014 Biophys. J. paper [13] by Keener and Shtylla suggests that kinetochore component flexibility and microtubule shape variation under load significantly reduces the need for weak specific binding of kinetochore components, and a paper by Powers et al. [14] demonstrated how an ensemble of Ndc80 complexes allows a kinetochore to maintain a load-bearing tip attachment to microtubules.

Although these papers are indirectly in accord with electrostatic forces acting at microtubule free ends, the paper by Miller et al. [4], advances Ndc80^Hec1^ as explicitly responsible for electrostatics-based force generation at kinetochores and is of primary interest in the present work. Since direct electrostatic interactions are involved, force production is not dependent on microtubule splaying to generate a power stroke. It is proposed there [4] that the force-producing interaction is electrostatic since an unstructured positively charged Hec1 tail cannot bind microtubules lacking negatively charged C-termini; specifically, they show that increasing the salt concentration from 100 to 200 mM KCl “abolished almost all microtubule binding of Hec1(1–230) and Hec1(1–80).” The Ndc80^Hec1^ subunit (Hec1) has a microtubule-interacting site that includes a calponin homology domain, and an unstructured positively charged N-terminal tail that is sufficient for microtubule binding in vitro [15–17]. A lock and key mechanism involving the calponin homology domain, which has been associated with kinetochore attachments to microtubule ends [18, 19], does not explain complex chromosome motions. Alternatively, we suggest that calponin may serve to position and stabilize the microtubule-kinetochore end-on attachment, while the highly positive, unstructured tail of Ndc80/Hec1 is likely the dynamic electrostatic link with microtubule ends. This is in agreement with Miller et al.: “…our data argue strongly that the Hec1 tail is the critical attachment for depolymerization-coupled movements of chromosomes.”

They also conclude that their data shows that “…the highest affinity interactions between kinetochores and microtubules are ionic attractions between two unstructured domains”. Likewise, previous experiments involving the effect of calcium concentration [Ca^2+^] on Anaphase-A motions [20] constituted direct experimental evidence of an electrostatic basis for poleward force production [2]. This, in somewhat different language, i.e., involving electrostatic interactions between bound negative charge at microtubule plus ends and bound volume positive charge at kinetochores, resulted in essentially the same experimental outcome, as outlined below.

An optimum Ca^2+^ concentration for maximizing the speed of chromosome motions is observed during anaphase-A. If [Ca^2+^] is increased to a micromolar level, anaphase-A chromosome motion is increased two-fold above the control; however, if the concentration is further increased beyond a few micromolar, the chromosomes will slow down, and possibly stop [20]. It has long been recognized that one way elevated [Ca^2+^] could increase the speed of chromosome motion during anaphase-A is by facilitating microtubule depolymerization [21–25] which, if not the underlying force for chromosome poleward motions, is thought to be at least the rate determining step [26–29]. However, the slowing or stopping of chromosome motion associated with moderate increases beyond an optimum [Ca^2+^] is more difficult to interpret since the microtubule network of the spindle is not compromised to the extent that anaphase-A chromosome motion could be slowed or stopped; this would require considerably higher concentrations [20, 30]. Experimental observations of slowing or stopping of Anaphase-A motion are a direct consequence of higher concentrations of calcium ions screening the negative charge at the free ends of disassembling kinetochore microtubules, thus shutting down the poleward-directed nanoscale electrostatic disassembly force at the kinetochore. Since this happens at Ca^2+^ concentrations that do not compromise the spindle’s microtubule network, these observations are experimentally consistent with a poleward electrostatic microtubule disassembly force at kinetochores. It follows that shielding of negative charge at free microtubule plus ends by calcium ions [2] would have the same effect on microtubule plus ends as increasing the salt concentration from 100 to 200 mM [4].

Force generation at kinetochores has emerged as one of the signature problems in mitotic movements. With an abundance of proposals regarding poleward force generation at kinetochores seeking consensus, how does one decide which approach is the most compelling? Regarding scientific models, the renowned physicist Paul Ehrenfest has suggested that they should be framed in such a manner that “the essence lies in recognizing the connections in all directions.” Here we propose that the agreement between the Ndc80/Hec1 experiments and a classical electrostatics model strengthens both because a wide range of experiments is consistent with this combination.

Recognizing the connections in all directions was the basis for framing a classical electrostatics model (CEM) to address mitotic chromosome motions [1, 2]. This anticipated the aforementioned molecular biology approach regarding Hec1 as “unstructured”—volume, in CEM terms—positive charge at kinetochores, as well as the subsequent experimental discovery that centrosomes are negatively charged [31]. We propose here that the electrostatic nature of the Hec1 force producing function at kinetochores is consistent with a larger role for electrostatics in mitosis. Specifically, an ab initio calculation of the maximum (tension) force per microtubule that agrees with observation will be given, further supporting the model of Miller et al. [4] for poleward force production at kinetochores.

In summary, the approach taken here supports the role of Hec1 as bound volume positive charge distributions—“positively charged Hec1 tails” [4], in molecular biology terminology—at kinetochores, interacting electrostatically with bound negative charge at and near the free ends of microtubules—“ionic attractions between two unstructured domains” [4] in molecular terminology—as the cause for poleward force generation at kinetochores. Electrostatic generation of poleward force at cell poles has been considered elsewhere [32, 33].

## Some cellular electrostatics

Chromosome movements depend on kinetochore-microtubule dynamics: a chromosome can move toward a pole only when its kinetochore is connected to microtubules emanating from that pole [34]. Microtubules continually assemble and disassemble, so the turnover of tubulin is ongoing. The characteristics of microtubule lengthening (polymerization) and shortening (depolymerization) follow a pattern known as “dynamic instability”: that is, at any given instant some of the microtubules are growing, while others are undergoing rapid breakdown. In general, the rate at which microtubules undergo net assembly—or disassembly—varies with mitotic stage [35].

In the cytoplasmic medium (cytosol) within biological cells, it has been generally thought that electrostatic fields are subject to strong attenuation by screening with oppositely charged ions (counterion screening), decreasing exponentially to much smaller values over a distance of several Debye lengths. The Debye length within cells is typically given to be of order 1 nm [36], and since cells of interest in the present work (i.e. eukaryotic) have much larger dimensions, one would be tempted to conclude that electrostatic force would not be a major factor in providing the cause for mitotic chromosome movements in biological cells. However, the presence of microtubules, as well as other factors to be discussed shortly, changes the picture completely.

Microtubules can be viewed as intermediaries that extend the reach of electrostatic interactions over cellular distances, making the second most potent force in the universe available to cells in spite of their ionic nature. Microtubules are 25 nm diameter cylindrical structures comprised of protofilaments, each consisting of tubulin dimer subunits, 8 nm in length, aligned end-to-end parallel to the microtubule axis. The protofilaments are bound laterally to form a cylindrical microtubule. Cross sections reveal that the wall of a microtubule consists of a circle of 4 to 5 nm diameter subunits. The circle typically contains 13 subunits as observed in vivo. Neighboring dimers along protofilaments exhibit a small (B-lattice) offset of 0.92 nm from protofilament to protofilament. A number of investigations have focused on the electrostatic properties of microtubule tubulin subunits [37–40]. Large-scale calculations of tubulin have determined the dipole moment to be as large as 1800 Debye [38, 41]. Experiments [42] have shown that tubulin net charge depends strongly on pH, varying quite linearly from −12 to −28 (electron charges) between pH 5.5 and 8.0. This may be critical during mitosis because a number of cell types exhibit a decrease of 0.3–0.5 pH units from a peak at prophase during mitosis [43]. At pH 7, tubulin has a large overall charge of −20, and up to 40 % of this charge resides on C-termini [44]. The C-termini can point nearly perpendicularly outward from the microtubule axis as a strong function of pH_i_, extending 4–5 nm at pH_i_ 7 [44], and can exist in at least 2 other conformational states where they bind to the microtubule surface at lower pH_i_ [45].

As will be discussed below, quite apart from the ability of microtubules to extend electrostatic interactions over cellular distances, the range of electrostatic fields within the cytosol itself is longer than ordinary counterion screening considerations would dictate. Consequently it is likely that the electric dipole nature of tubulin subunits greatly assists in their self-assembly into the microtubules of the asters and spindle. Thus dipole electrostatic fields organize and align electric dipole dimer subunits, thereby facilitating their assembly into microtubules that form the asters and the mitotic spindle [46]. This self-assembly is aided by reduced counterion screening due to layered water adhering to the net charge of the dipolar subunits. Such water layering to charged proteins has long been theorized [47, 48], and confirmed experimentally [49]. Additionally (see below), layered water between closely spaced, charged proteins has a dielectric constant that is considerably reduced from the bulk value far from charged surfaces, further increasing the tendency for electrostatically–assisted aster and spindle self-assembly.

The combination of water layering and reduced dielectric constant can significantly influence cellular electrostatics in a number of important ways related to cell division. For clarity, gaps between charged surfaces within cells that allow these two effects to enhance electrostatic interactions will be referred to as critical gaps or critical distances. These two conditions for charged molecular surfaces at close range will also be seen (“Electrostatic microtubule poleward disassembly force at kinetochores” section) to have important consequences regarding force generation for poleward motion of chromosomes during mitosis. An electrostatic component to the biochemistry of the microtubules in assembling asters is consistent with experimental observations of pH effects on microtubule assembly [50], as well as the sensitivity of microtubule stability to calcium ion concentrations [21].

Considering the electrostatic properties of tubulin dimers, an increasing microtubule disassembly to assembly probability ratio—with attendant changes in microtubule dynamics and associated chromosome motions through metaphase—can be ascribed to an experimentally-observed steadily decreasing pH_i_. Thus a decrease in pH_i_ from a peak at prophase favoring microtubule assembly, declining through prometaphase, and continuing to decline through metaphase when parity between microtubule assembly and disassembly leads to midcell chromatid pair oscillation, culminating in the observed increased microtubule disassembly-associated kinetochore tension late in metaphase is likely integral to changing chromosome movements.

Stated differently, a decrease in pH_i_ through mitosis may act as a master clock controlling microtubule disassembly/assembly probability ratios by altering the electrostatic interactions of tubulin dimers. This, in turn, would determine the timing and dynamics of post-attachment chromosome motions through metaphase [51].

As mentioned above, electrostatic poleward force generation at cell poles mirroring the present work is discussed elsewhere [32, 33]. Because of the similarity to the calculation in "Electrostatic microtubule poleward disassembly force at kinetochores" section, this will be briefly outlined here as another aspect of a CEM approach to mitotic motions. Given negatively charged centrosomes [31], and positive charge at the minus ends of microtubules at centrosomes [32], one can envision a mirror-image situation similar to that at kinetochores. The electric field of a centrosome will attract and draw positively charged minus ends of microtubules into the centrosome. The changing electric field (and resulting force) gradient vicinal to, and across, the centrosome matrix boundary would destabilize microtubules, thus increasing the depolymerization probability of microtubules approaching and penetrating a centrosome as force is generated, in agreement with experiment.

## Electrostatic microtubule poleward disassembly force at kinetochores

**Fig. 1:**
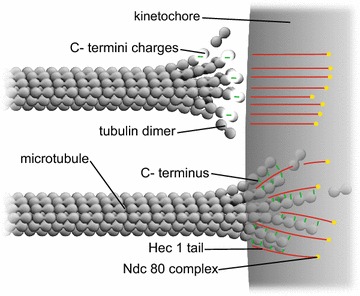
Nanoscale electrostatic disassembly force acting at a small section of a kinetochore. The *top* microtubule depicts electrostatic interactions over critical distances between groups of negative charges on C-termini near microtubule free ends and positively charged Hec 1 tails. The *bottom* microtubule depicts the interactions between negatively charged C-termini and positively charged Hec 1 tails within a kinetochore

Here we calculate the poleward force at kinetochores due to both penetrating and non-penetrating microtubule plus ends. As mentioned above, the calculations are applicable to—and supportive of—the paper by Miller et al. [4]. A small section of a kinetochore interacting with several microtubules is depicted in Fig. 1. Accordingly, within the context of the present work, poleward force generation at kinetochores for prometaphase post-attachment, metaphase, and anaphase-A poleward chromosome motions can be attributed to an electrostatic attraction between the negatively charged free plus ends of kinetochore microtubules and a positively charged kinetochore.

A calculation of the magnitude of the poleward force produced in this manner by a non-penetrating microtubule at a kinetochore follows. Since the outer plate diameter of a kinetochore is somewhat larger than the diameter of a protofilament, it will be modeled as a large, approximately planar slab with positive surface charge density of magnitude *σ* as depicted in Fig. 1. This modeling is completely consistent with typical experimental values of kinetochore diameters of 100’s of nanometers. For example, values for PtK1 cell kinetochore radii range from 160 to 230 nm (0.16 ± 0.05 µm^2^ kinetochore area) [52], as compared to 4–5 nm for protofilament diameters. From the well-known Debye-Hückel result for a planar charged surface with area charge density *σ* immersed in an electrolyte [53], the electrostatic potential is:

1 $$\varphi \left( x \right) = \left( {{{D\sigma } / \varepsilon }} \right)e^{{{{ - x} / D}}}$$

where *D* is the Debye length, *ε* is the cytosolic permittivity (*ε* = *k ε*_0_, with *k* the dielectric constant, *ε*_0_ the permittivity of free space), and *x* the distance from the surface.

The electric field *E*(*x*), obtained from the negative gradient of the electrostatic potential, multiplied by the charge *q* gives the magnitude of the attractive force *F* (*x*) between the charge *q* on a dimer subunit at the free end of a protofilament and the kinetochore. This results in

2 $$F\left( x \right) = qE\left( x \right) = - q\left( {{{\partial \varphi \left( x \right)} / {\partial x}}} \right) = \left( {{{\sigma q} / \varepsilon }} \right)e^{{{{ - x} / D}}}.$$

It is well established in electrochemistry [54] that the permittivity of the first few water layers outside a charged surface is an order of magnitude smaller than that of the bulk phase. The effective permittivity of water as a function of distance from a single charged surface has been determined by atomic force microscopy [55] to increase monotonically from 4 to 6 *ε*_0_ at the interface to 78 *ε*_0_ at a distance of 25 nm from the interface. The values of the dielectric constants *k*(*x*) at distances of 1, 2, 3, and 4 nm from a charged surface were measured to be 9, 21, 40, and 60, respectively.

The interpolated values of *k*(*x*) for separations between charged surfaces of up to 3 nm are 5, 9, 9, and 5 for *x* = 0, 1, 2, and 3 respectively, where the charged surfaces are at *x* = 0 and *x* = 3 nm (the experimental value of *k*(*x*) at both *x* = 0 and *x* = 3 is 5, and symmetry and the experimental numbers dictate the values of 9 in between.) The distance range 1–3 nm between charged surfaces is significant for the present calculation because 1 nm may be taken as the thickness of layered water adsorbed to each charged surface [48, 56], and for charged molecular surface separations up to 3 nm, counterion (Debye) screening would be virtually eliminated. Thus electrostatic force is increased over the distances allowed by reduced Debye screening, and is further increased (by an order of magnitude) due to an order of magnitude reduction in the dielectric constant between the charged surfaces. For brevity, separations of 0–3 nm (and—due to the reduced dielectric constant between charged molecular surfaces 1 to 2 nm beyond) between charged surfaces will hereafter be designated as *critical distances/gaps*.

For critical distances, the expression for the magnitude of the force between a charged kinetochore surface at *x* = 0 and a charge *q* on the free plus end of a protofilament at a distance *x* from the surface may therefore be written

3 $$F\left( x \right) = {{\sigma q} / {\varepsilon \left( x \right)}}$$

where *ε*(*x*) = *k*(*x*) *ε*_0_ is obtained from the interpolated experimental results for *k*(*x*) referred to above, *ε*_0_ = 8.85 pF/m (picoFarads per meter) and *q* is the charge on the protofilament free end. This equation may be obtained from (2) in the limit as *D* → ∞, a condition that effectively eliminates counterion screening.

There are 13 protofilaments arranged circularly in a microtubule, with an axial shift of 0.92 nm for each protofilament as one moves around the circumference of a B lattice microtubule [38]. For comparison with experimental values, and to get a sense of the strength of the electrostatic forces, a calculation of the total disassembly force per microtubule due to protofilaments at distances of 2 and 3 nm from a kinetochore will be carried out. The actual distribution for the distances of free ends of 13 disassembling (curling), and temporarily assembling (straight) protofilaments would be considerably complicated, and most likely several protofilaments from a proximal microtubule will interact with a kinetochore within critical distances at any given time.

Older experimental values of surface charge density *σ* for biological surfaces range from 1 to 50 mC*/*m^2^ (milliCoulombs per square meter) [57, 58]. The N-terminal tail of Hec1 contains an equivalent net positive charge of ten (electron charges) [4]. In addition, kinetochores contain at least eight Hec1 proteins per microtubule [59], giving a possible 80 charge interactions per microtubule, or 1600 (20 × 80) per human kinetochore [4]. For a human kinetochore area of 250,000 nm^2^ [60], this is equivalent to a positive area charge density of 0.006 e*/*nm^2^ (electron charge magnitudes per square nanometer), converting to 1.1 mC*/*m^2^. Thus, we may calculate the forces on protofilament free ends at the above distances from a kinetochore using the interpolated *k*(*x*) values of 9 at the 2 nm distance and 5 at the 3 nm distance, along with the value for *σ* of 1.1 mC*/*m^2^. Carrying out this calculation with (3), we find that the electrostatic force on the two protofilaments sums to 6 *n* pN/MT (picoNewtons per microtubule), where *q* = *ne*, with *e* equal to the magnitude of the charge on an electron and *n* the number of electron charges at the protofilament free end. Comparing this value with the experimental range of 1–74 pN/MT [61] for the maximum tension force per microtubule, we have that *n* = 0.17–12 electron charges. Recent tension force measurements [62] have set the high end of the above range to a few pN/MT, indicating that the lowest values of *n* are more likely. Based on the more recent experiments, a range of 1–5 pN/MT results in *n* = 0.17–0.83 electron charges. This range of values compares favorably to experiments [38, 40, 63], and the agreement represents a successful ab initio theoretical derivation of the magnitude of this force. Note that this calculation, like the others in this paper, can be done in a number of ways dependent on the specific assumptions. However, all of the justifiable calculations lead to ranges for protofilament free end charges that are well within the experimental range.

Thus force generation from an instantaneous subset of protofilaments (at critical kinetocore distances within a number of microtubules) continues with other subsets of constantly changing larger, and smaller (critical) gaps, causing kinetochore microtubule bundles to move toward a kinetochore—with kinetochores moving poleward—while doing work. Polymerization in gaps larger than the 8 nm length of tubulin dimers, along with depolymerization elsewhere, continues as overall “contact”/tracking is maintained by critical gap forces. Importantly, nanoscale electrostatic forces acting at critical distances can maintain overall contact/tracking throughout the complex motions of mitosis. Note that polymerization in gaps slightly greater than 8 nm would be expected to place tubulin dimers close to or within critical distances for force generation. With an increase in the microtubule disassembly to assembly probability ratio (higher net disassembly rate), there will be less opportunity for polymerization since advancing microtubules can more frequently shorten kinetochore distances to less than 8 nm.

We now proceed to calculate the electrostatic force at a kinetochore due to penetrating microtubules. Since kinetochore diameters are large compared to the diameters of protofilaments, we may model the kinetochore-microtubule interaction for penetrating microtubules by assuming an approximately planar slab of uniform volume positive charge density, with thickness *a* parallel to the *x* axis (the microtubule axis) for the outer “plate” of a kinetochore, interacting with negatively charged microtubule protofilaments, as depicted in Fig. 1.

A standard result from an application of Gauss’s law [64] gives the following result for the magnitude of the electric field inside a large (compared to the thickness *a*), uniformly charged slab of positive charge

4 $$E\left( x \right) = \rho x/\varepsilon_{1}$$

where *ρ* is the volume charge density, *ε*_1_ (= *k*_1_ *ε*_0_) is the dielectric permittivity of the slab, and *x* = 0 at the plane of symmetry in the center of the large rectangular slab. (Note that previously in (3), *x* = 0 at the left boundary of the kinetochore, Fig. 1).

Making use of the uniform charge relation *σ* = *ρa*, this result may be expressed in terms of the surface charge density *σ* as

5 $$E\left( x \right) = \sigma x/\varepsilon_{ 1} a$$

The magnitude of the force on a protofilament of negative charge magnitude *q* at its free end a distance *x* from the plane of symmetry is given by

6 $$F\left( x \right) = qE\left( x \right) = q\sigma x/\varepsilon_{1} a$$

At the left boundary of the (positively charged) kinetochore, *x* = −*a*2, *E* = −*σ*/2*ɛ*_1_, and the magnitude of the force exerted in the positive *x* direction (with an equal and opposite poleward force on the kinetochore) on a protofilament free end with negative charge of magnitude *q* at its free end located just inside the left face is *σq*/2*ɛ*_1_.

The value of the dielectric constant *k*_1_ for a kinetochore has not been established. Due to an open structure that allows cytoplasmic water intrusion, the large dielectric constant of water would strongly influence the overall dielectric constant of the kinetochore, leading to a value that is relatively insensitive to the dry value. Consistent with their open structures, a cytosol-saturated kinetochore would be expected to have a dielectric constant that is quite large, roughly midway between the dry value and cytoplasmic water [65]. Therefore (1) the value for cytoplasmic water will dominate, and (2) the calculation is relatively insensitive to the precise dry value. For simplicity, since most condensed-matter dielectric constants are between 1 and 5, an approximate conservative midpoint value *k*_1_ = 45 [(80 + 10)*/*2] will be assumed.

Using *k*_1_ = 45 and the value *σ* = 1.1 mC*/*m^2^ in carrying out a conservative calculation with (6) for a microtubule with 6 of the 13 protofilament ends at an average distance just inside the left boundary (*x* = − *a/*2) of the kinetochore, we find that the force on a penetrating microtubule sums to 1.3 *n* pN/MT. Equating this result to the experimental range 1–5 pN/MT, we find that *n* = 0.77–3.8 electron charges, again within the experimental range.

Given the electrical nature of tubulin microtubule subunits, the electric field (and therefore force) gradient within vicinal cytosol at a kinetochore, as well as within a kinetochore, would increase the lability of microtubule minus ends. Additionally, the field gradient across the kinetochore-cytosol boundary can act to destabilize microtubules, increasing the depolymerization probability of microtubules approaching and penetrating a kinetochore as force is generated, which is in agreement with observations.

Antipoleward electrostatic forces for chromosome motions are also integrated into the complex motions of mitosis. As discussed elsewhere [33, 66], chromosome congression likely results from a combination of poleward force at kinetochores and poles with an inverse square antipoleward electrostatic microtubule assembly force acting at chromosome arms. The dominance of the inverse square dependence of antipoleward microtubule assembly forces over poleward microtubule disassembly forces is primarily responsible for chromosome congression, as well as metaphase chromosome oscillations. Chromosome end-on attachment orientation and the “slip-clutch” mechanism are also consistent with this combination of opposing forces [66].

## Conclusions

It seems clear that cellular electrostatics encompasses more than traditional thought regarding counterion screening of electric fields and disregarding the second most powerful force in nature. The reality is that experimental evidence suggests otherwise, and electrostatic interactions are more robust and act over greater distances than previously thought. One aspect of this realization is that microtubules extend the reach of electrostatic force over cellular distances; another lies in the reduced counterion screening and dielectric constant of the cytosol between charged molecular surfaces.

Given positive charge on kinetochores and negative charge at plus ends of microtubules, it is difficult to conceptualize there not being an attractive electrostatic poleward-directed force between these structures. Calculations of electrostatic force magnitudes for penetrating and non-penetrating microtubules show that nanoscale electrostatic interactions account for poleward force generation at kinetochores. These calculations fall within observed experimental ranges, thus representing a successful ab initio derivation of the magnitude of this force. The present model assumes that force generation is due to both penetrating and non-penetrating microtubules. Force generation by nanoscale electrostatic non-contact interactions, primarily over critical distances, is essential for efficient microtubule reattachment and tracking to kinetochores throughout the complex motions during mitosis.

Antipoleward force on chromosomes within a CEM demonstrates that combining electrostatic antipoleward microtubule assembly forces on chromosome arms with poleward microtubule disassembly forces at kinetochores as described here is sufficient to explain chromosome congression.

Changes in microtubule dynamics are integral to changes in chromosome motions during mitosis, and can be attributed to an associated change in intracellular pH within a CEM. A decrease in intracellular pH—from a peak at prophase through mitosis may act as a master clock controlling microtubule disassembly to assembly probability ratios and the associated motions of chromosomes by altering electrostatic interactions between tubulin dimers.

Polar generation of poleward force is addressed within a CEM by a calculation that mirrors the present work.

Current literature regarding mitotic chromosome post-attachment motility focuses primarily on poleward generation of force at kinetochores. Clearly this is a small part of a broader question regarding the complex motions of chromosomes throughout mitosis. Our goal here is to show that a CEM for poleward force generation at kinetochores supports ionic attractions between two unstructured molecular domains at kinetochores leading to poleward force production while being consistent with experiments encompassing a wider spectrum of mitotic movements.

## Abbreviation

CEM: classical electrostatics model
