# Erratum to: Electrostatic forces drive poleward chromosome motions at kinetochores

**DOI:** 10.1186/s13008-017-0030-0

**Published:** 2017-07-03

**Authors:** L. John Gagliardi, Daniel H. Shain

**Affiliations:** Departments of Physics and Biology, Rutgers The State University of New Jersey, Camden, NJ 08102 USA

## Erratum to: Cell Div (2016) 11:14 DOI 10.1186/s13008-016-0026-1

A primary objective of our paper [1] is to provide numerical support for an approach to poleward force generation for chromosome motility that is based on electrostatic attractions between bound cellular charge distributions [2]. This involves electrostatic interactions between negative charges on C-termini at free ends of microtubules and unstructured positively charged Hec1 tails as depicted in Fig. 1 of our paper. Figure 1 is integral to the approach taken in our paper, but it does not elucidate the force-producing mechanism for kinetochore-penetrating microtubules. The purpose of this note is to provide a figure that explicitly shows the electrostatic force-producing mechanism proposed here.

**Fig. 1:**
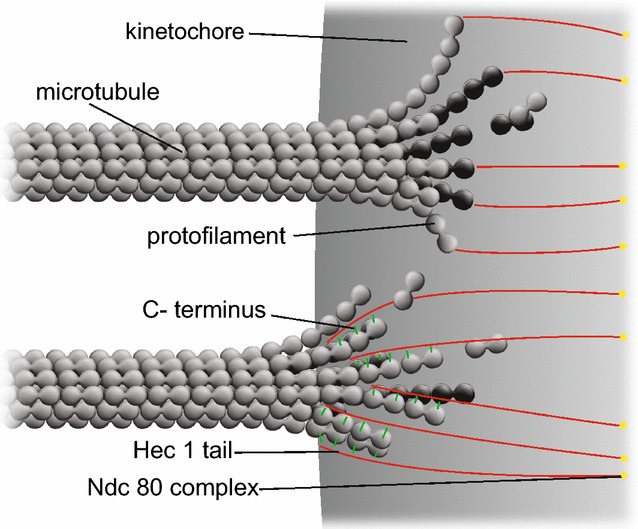
**a** Nanoscale electrostatic disassembly forces acting at a small section of a kinetochore. The *bottom* microtubule depicts electrostatic interactions between negatively charged C-termini on gently curving protofilaments and positively charged Hec1 tails. The *top* microtubule depicts a possible configuration at the point of release between previously interacting C-termini and Hec1 tails. Because of the geometry effect of greater protofilament curvature at a later stage of a progressively-splaying microtubule, the loss of proximity of C-termini charges on concave sides of protofilaments to Hec1 tails leads to a failure of the electrostatic interactions

In particular, since C-termini are on the outsides of microtubules, as the curvature of splaying microtubule protofilaments increases, the electrostatic attractions between charges on Hec1 tails and C-termini will weaken and fail because of the loss of close proximity between the two charge distributions.

This situation is detailed in Fig. 1a, which shows a time sequence between force-generating gently splaying protofilaments, and the later failure of these protofilaments to interact electrostatically because of the geometry effect of increased protofilament curvature.

As depicted in the top microtubule in Fig. 1a, since C-termini are on the concave sides of progressively splaying microtubules, increasing protofilament curvature will lead to a separation of the charges on Hec1 tails and C-termini. Accordingly, subsets of low-curvature splaying protofilaments generate poleward force, while other subsets of protofilaments with more pronounced curvature in later stages of depolymerization will fail to bind. Thus poleward force is generated as microtubules depolymerize, in agreement with observation.
